# Can patients who apply to tertiary care with headache for the first time be managed in primary care? - a cross-sectional study

**DOI:** 10.3906/sag-2010-237

**Published:** 2021-08-30

**Authors:** Esra HÜNDÜR DOĞAN, İrfan ŞENCAN, Hasan DOĞAN, Adem ÖZKARA

**Affiliations:** 1 Department of Family Medicine, University of Health Sciences, Ankara City Hospital, Ankara Turkey; 2 Department of Neurology, University of Health Sciences, Ankara City Hospital, Ankara Turkey

**Keywords:** Primary care, headache, family physician

## Abstract

**Background/aim:**

Headaches are one of the most common neurological symptoms. They adversely affect daily life, reduces the workforce, and have high health costs. Managing this symptom in primary care will free up secondary and tertiary health services to better treat patients who need follow-up by specialists. In the present study, we aim to show the rate at which this problem can be solved in primary care for patients who applied tertiary care services with a headache for the first time.

**Materials and methods:**

Our research is a cross-sectional study of 207 patients who applied to the neurology clinics for the first time with headache. Two questionnaires were prepared by the researchers. IBM SPSS v: 21.0 was used for statistical analysis, and the level of significance was p < 0.05.

**Results:**

The opinions of the patients on the evaluability of headache in primary care were compared. Family physicians and neurologists gave similar responses about the disease management, at a rate of 96.6%, this was found to be statistically significant and shows strong agreement. Both groups of physicians thought that 70% of patients, on average, who applied to the neurology clinics with headache for the first time could be managed in primary care. However, only 9.2% of the patients share this opinion with physicians. Primary headache cases constitute most of the cases that are thought to be solved in primary care. It was revealed that the number of patients seeking primary care with this complaint was lower than expected.

**Conclusion:**

Patients with headache applied tertiary care instead of primary care for different reasons. Both neurologists and family physicians stated that most patients evaluated in tertiary care could be managed in primary care.

## 1. Introduction

Headache, one of the most common reasons for consulting a physician, is a social and economic problem that causes a decrease in the quality of life, an increase in the level of incapacity to work of individuals, and an increase in health expenditures. It is the health problem that physicians most frequently encounter and it often affects patients and their relatives. 

According to the International Headache Society, headaches are categorized as primary, secondary, neuropathy, and other [1]. Primary headaches comprise 90% of headaches [2]. 

“Red flags,” which are significant issues to be considered in secondary headaches, should be well known. Anamnesis is the most important element in diagnosing a patient with a headache. The first step in evaluating complaints correctly is to differentiate between primary and secondary headaches. With a correct anamnesis, most patients can be diagnosed, treated, and referred to the appropriate specialty. 

The percentage of people who have experienced a headache at least once in their lifetime is above 90% [3]. It is one of the top ten reasons to see a family doctor [4]. Other common reasons that may lead to secondary headaches are acute/chronic sinusitis, ear infections, and hypertension [5]. At neurology outpatient clinics, two-thirds of patients have headache complaints. One-third go to neurology outpatient clinics with only headache complaints [6].

It would be best for the first evaluation of headache patients to be done by the family physician, who knows the patient better, is more available, and can take a holistic approach. This may also help prevent polypharmacy and unnecessary advanced examinations. For the health system to function well, it is important to evaluate headache complaints that can be managed in primary care, since there are so many headache patients in neurology outpatient clinics. These clinics offer secondary and tertiary care with little time per patient and transportation challenges. Managing headaches in primary care will enable specialists to do better follow-up with patients who do need secondary and tertiary care. 

In light of this, we aimed to determine the rate at which patients who went to the neurology outpatient clinic of Ankara City Hospital for the first time with headache complaints could be followed up and treated in primary care. Furthermore, we aimed to reveal the differences in disease management by asking the opinions of branch physicians, family physicians, and patients. 

## 2. Materials and methods

Our research is a cross-sectional study. The study population consists of 207 patients who were older than 18 years of age who were admitted to the neurology outpatient clinics of Ankara City Hospital for the first time between 01.02.2020 and 31.03.2020 with headache complaints. In five outpatient clinics, there are on average 60 patients per day. During our study, a pandemic was declared due to the COVID-19 outbreak, and the number of active outpatient clinics was reduced to two and the average number of patients was 25. The number of patients with headache applied for the first time, which was 6–7 after this date, has decreased to 2–3. To calculate the number first-time headache patients we used the G-Power 3.1.5 program. The minimum sample size was calculated to be 172 with a medium effect size of 0.05-α error probability, 0.95 power (1-β error), and two degrees of freedom for the goodness of fit test. 

The data collection process was started after obtaining approval E-19-204 from Ankara City Hospital No. 1 Clinical Research Ethics Committee on 24.12.2019. Our study was conducted in accordance with the Declaration of Helsinki. 

Informed consent was obtained from all patients before starting the questionnaire. When patients were called to the examination room, they were first asked about their symptoms, and they had headaches, and if this was their first time at the clinic. The admission number of the patients who applied for the first time was written in the “patient number” part of the questionnaire. Imaging examinations were categorized at the patient’s and physician’s request, and after the appropriate category was marked, the imaging method was noted. Afterward, it was noted which International Statistical of Diseases and Related Health Problems (ICD) code was used by the physician for the diagnosis/pre-diagnosis. The neurologist’s recommendation was noted: follow-up, control, examination, medication, or consultation. After the examination of the patient was completed, the assistant researcher filled in the ‘family physician researcher’s opinion’ on whether the patient could be evaluated in primary. The ‘neurologist’s opinion’ section of the questionnaire was directed to the relevant branch physician, and one of the options was marked: yes, no, or not sure. After the patient left the room, the patient’s informed consent was received and the patient was asked if the headache could be treated in primary care, and the second form was used. It took approximately two minutes to fill out the questionnaire.

The main groups we compare are the patient group with primary and secondary headache. The multiple groups we compare are the opinions of family physicians, neurologists, and patients on whether headache can be evaluated in primary care.

Statistical analysis was conducted using IBM SPSS v: 21.0 (IBM Corp., Armonk, NY, USA). Descriptive statistics are presented as a number and percentage. Measurement data were evaluated with the Kolmogorov–Smirnov test and histograms for compliance with normal distribution and are presented with mean and standard deviation values ​​because they have normal distributions. The chi-square test and Fisher’s exact test were used to compare count data in the statistical evaluation and the Student’s t-test was used in paired groups because they had normal distributions. The Bonferroni correction was applied to detail the significance between the categories and determine which groups had a relationship. The consistency between the two approaches was examined by Cohen’s Kappa test. Type I error level was accepted at 0.05. 

## 3. Results

The mean age of the 207 patients was 40.98 ± 13.41 years; 65.2% were female and 34.8% were male. Most patients had state health insurance (SHI), were high school graduates, and lived in the city center (Table 1).

**Table 1:** Sociodemographic characteristics of the participants.

		Total		Primary HA˚		Secondary HA		
		n	%	n	%	n	%	P*
Age group	18–34	73	35.3	49	67.1	24	32.9	0.001
	35–49	76	36.7	40	52.6	36	47.4	
	50–64	48	23.2	19	39.6	29	60.4	
	65+	10	4.8	1	10.0	9	90.0	
Sex	Female	135	65.2	78	57.8	57	42.2	0.043
	Male	72	34.8	31	43.1	41	56.9	
Educational status	Illitare	15	7.2	7	46.7	8	53.3	0.899
	Primary education	69	33.3	36	52.2	33	47.8	
	High school	83	40.2	46	55.4	37	44.6	
	University and above	40	19.3	20	50.0	20	50.0	
Social security	SHIⁱ	198	95.7	103	52.0	95	48.0	0.627
	Private	4	1.9	3	75.0	1	25.0	
	Foreign nationality	4	1.9	2	50.0	2	50.0	
	None	1	0.5	1	100.0	0	0.0	
Place of residence	Province	122	58.9	70	67.4	52	42.6	0.171
	District-village	73	35.3	35	47.9	38	52.1	
	Out of the province	12	5.8	4	33.3	8	66.7	

*Chi-square test, ˚HA: Headache, SHIⁱ: State health insurance.

When the patients were classified according to the headache category, 52.7% of the patients had a primary headache and 47.3% had a secondary headache. When examined according to the specific classification, the most common type of primary headache was a tension headache and the most common secondary headache was a headache due to diseases of the face and head structures (Table 2). 

**Table 2:** Headache diagnosis classification of the participants.

		n	%
Primary headaches	Total	109	52.7
	Tension-type headache	47	43.1
	Migraine	45	41.3
	Cluster headache and other trigeminal autonomic headaches	13	12.0
	Other primary headaches	4	3.6
Secondary headaches	Total	98	47.3
	Related to diseases of the face and head structures	20	20.4
	Due to the effect or discontinuation of medications	13	13.3
	Due to psychiatric diseases	8	8.2
	Due to homeostasis disorder	8	8.2
	Due to cranial or cervical vascular diseases	5	5.1
	Other	44	44.8

The attitudes and behaviours of the participants regarding their examination and results are summarized in Table 3. Only 58.0% of patients had gone to their family physician before. When the reasons for this were examined, 30.8% of the patients stated that they did not have family physicians or did not know the family physician.

**Table 3:** The comparison of the attitudes, behaviours, examination, and result characteristics of the participants regarding their application to family medicine and headache diagnostic categories.

		Total		Primary HAi		Secondary HA		
		n	%	n	%	n	%	p
Previous application to FP*	Yes	120	58.0	64	53.3	56	46.7	0.819
	No	87	42.0	45	51.7	42	48.3	
FP recommendation	LSC** + follow-up	9	7.5	7	77.8	2	22.2	0.275
	Medication	79	65.9	39	49.4	40	50.6	
	Advanced examination	22	18.3	11	50.0	11	50.0	
	Referral	10	8.3	7	70.0	3	30.0	
Implementing the FP’s recommendations	Yes	88	.9	45	51.1	43	48.9	0.755
	No	4	3.4	2	50.0	2	50.0	
	Partially	27	22.7	16	59.3	11	40.7	
Being satisfied with FP’s recommendations	Yes	27	22.7	15	55.6	12	44.4	0.067
	No	29	24.4	10	34.5	19	65.5	
	Partially	63	52.9	38	60.3	25	39.7	
The reason for not applying to FP	I do not have/I do not know a family physician	28	30.8	14	50.0	14	50.0	0.884
	Examination facilities are insufficient	26	28.6	13	50.0	13	50.0	
	I do not think he can solve my problem	27	29.7	16	59.3	11	40.7	
	Because he thinks more attention to him will be paid in the hospital/ Hospital staff	10	10.9	5	50.0	5	50.0	
Laboratory request	Is checked at FHC◦	137	95.8	73	53.3	64	46.7	0.012
	Cannot be checked at FHC	6	4.2	0	0.0	6	100.0	
Imaging request	Patient’s request	34	25.2	24	70.6	10	29.4	<0.001
	Physician’s request	101	74.8	36	35.6	65	64.4	
Imaging method	MRIa	122	90.4	59	48.4	63	51.6	0.005
	CTb/DOPPLER/EEGd	13	9.6	1	7.7	12	92.3	
Result	Control/follow-up	11	5.3	0	0.0	11	100.0	<0.001
	Examination	66	31.9	25	37.9	41	62.1	
	Medication	90	43.5	81	90.0	9	10.0	
	Consultation	40	19.3	3	7.5	37	92.5	

*FP: Family physician, **LSC: Lifestyle change, iHA: Headache, oFHC: Family health center, aMRI: Magnetic resonance imaging, bCT: Computed tomography, dEEG: Electroencephalography.

The relationship between the family physicians’, neurologists’, and the participants’ opinions regarding the evaluability of headache in primary care and the diagnostic categories is detailed in Table 4. In Table 5, the evaluability status of the patients in primary care is compared. There was strong agreement between neurologists and family physicians. 

**Table 4:** The relationship between the opinions of family physicians, neurologists and patients on the evaluability of the headache in primary care and the diagnostic categories.

		Total		Primary HAi		Secondary HA		
		n	%	n	%	n	%	p*
FP*s’ opinion	Yes	148	71.5	96	64.9	52	35.1	<0.001
	No	40	19.3	7	17.5	33	82.5	
	Not sure	19	9.2	6	31.6	13	68.4	
Neurologists’ opinion	Yes	143	69.1	92	64.3	51	35.7	<0.001
	No	54	26.1	14	25.9	40	74.1	
	Not sure	10	4.8	3	30.0	7	70.0	
Patients’ opinion	Yes	19	9.2	14	73.7	5	26.3	0.004
	No	107	51.7	45	42.1	62	57.9	
	Not sure	81	39.1	50	61.7	31	38.3	

i: Headache, *: Family physician.

**Table 5:** The comparison of the evaluability statuses of the patients in primary care with each other.

		FP*’s opinion						Patient’s opinion					
		Yes		No		Not sure		Yes		No		Not sure	
		n	%	n	%	n	%	n	%	n	%	n	%
Neurologist’s opinion	Yes	143	96.6	0	0.0	0	0.0	19	100.0	51	47.7	73	90.2
	No	3	2.0	40	100	11	57.9	0	0.0	49	45.8	5	6.1
	Not sure	2	1.4	0	0.0	8	42.1	0	0.0	7	6.5	3	3.7
		ĸ: 0.829 p < 0.001						ĸ: 0.141 p < 0.001					
FP’s opinion	Yes	-	-	-	-	-	-	19	100.0	56	52.4	73	90.2
	No	-	-	-	-	-	-	0	0.0	39	36.4	1	1.2
	Not sure	-	-	-	-	-	-	0	0.0	12	11.2	7	8.6
ĸ: 0.141 p < 0.001													

*:Family physician.

We compared the sociodemographic characteristics of the patients, the reason for going to the family physician, if they followed physician’s recommendations, and their satisfaction with the recommendations. Females went to the family physician more than males, they followed recommendations more often, and were more satisfied with the recommendations.

The specialty of the family physician positively affected the applications, the implementation, and satisfaction with the recommendations. Of the participants whose family physician had a specialty, 91.5% had previously been to the family physician with a headache and 67.3% of the participants whose family physician did not have a specialty had previously applied to the family physician with a headache. 

The sociodemographic characteristics of the participants and the family physician’s opinion on the evaluability of the patient in primary care were compared. The family physician researcher thought that compared to other age groups, patients over 65 years of age who seek care with headaches are less likely to be evaluated in primary care. This is also statistically significant. The family physicians stated that the headaches of participants from outside the province could be evaluated in primary care at a statistically significantly lower rate in comparison with the other groups. 

## 4. Discussion

Although most of the patients included in the study had a primary headache according to the headache diagnostic classification, the rate was lower than reported in the literature [7,8]. We think that the reason for this is that secondary headache disorders are more frequent, require faster diagnosis and treatment, and can cause more serious problems during the COVID-19 pandemic. 

More than half of the patients included in the study stated that they had previously consulted their family physician mostly to acquire an analgesic prescription. In a study conducted by Durmuş et al., the rate of seeking primary care before hospital care for headaches was 24.6% [9]. In the dissertation of Ayazoğlu, prescribing was in the first place among the reasons for applying to family health centers [10]. In our study, the rate of analgesic use was high and the rate of referral to the family physician of patients with headache was higher than in the general population. We think that the reason for this is that people go to primary care to acquire analgesics rather than to be diagnosed. 

Patients with headaches may be advised to avoid triggers and make lifestyle changes. Positive changes in the main etiology of secondary headache causes may be obtained with appropriate recommendations [11,12]. In the present study, it was notable that the rate of recommending lifestyle changes was quite low. We think that family physicians, who are one of the most important elements of preventive health services and who know patients better, should recommend lifestyle changes more frequently. 

The questionnaires showed that most patients followed doctors’ recommendations but fewer patients were satisfied with the recommendations. In a study carried out by Berberoğlu et al., 82.8% of patients were satisfied with treatments by family physicians [13]. Another study by Durmuş et al. had a similar result, 80.7% [9]. On this issue, our study including partially satisfied agrees with the literature. 

The reasons for not going to a family physician are examined were that the patients do not know the family physician or did not have one. In the study by Durmuş et al., 15.1% of individuals did not know their family physicians [9]. The reason for the high rate in our study may be that it was performed among patients who were admitted to a tertiary care hospital and that foreign patients seek tertiary care more frequently than primary care. Another reason was that the examination facilities were regarded as insufficient. Güven et al. showed that 78.4% of patients regarded family health center facilities as insufficient. In the same study, 47.7% of the patients found the knowledge level of their family physicians insufficient and 28.4% thought that their problems could not be solved [14]. These rates are similar to those in our study. Since there was hospital staff, at a rate of 10.9% in our study, there were patients who could not seek primary care. This is due to the fact that the hospital in which the study was conducted has a high number of personnel and these personnel cannot leave the institution where they work during working hours. 

Laboratory examinations were requested by the neurologist in the majority of the patients, which showed that most of them could be examined in primary care. It was statistically significant that all cases that could not be examined in primary care were secondary headaches. 

One-quarter of the patients made imaging requests, mostly by patients with primary headache. In the study by Ay et al., 92.9% of the magnetic resonance imaging (MRI) results in headache patients were normal [15]. In the presence of alarm symptoms, an imaging method should be requested as well as a detailed history and neurological examination [16]. We think that imaging may have been requested because the patients requested it, in order to avoid malpractice and because imaging methods were available, since the study was performed in a tertiary care hospital [17]. 

Treatments administered to patients and resulting in medication were mostly given for prophylaxis and included beta-blockers, amitriptyline, selective serotonine reuptake inhibitor, serotonine norepinephrine reuptake inhibitor, flunarizine (calcium channel blocker), and topiramate. Simple analgesics, nonsteroidal antiinflammatory drugs (NSAIDs) were used most commonly as analgesic treatment. Medications administered by the neurologist can also be prescribed by the primary care physician, who can also arrange the necessary treatment for prophylaxis, especially for primary headaches. By applying the appropriate treatment for prophylaxis, the frequency of attacks can be reduced, workforce loss can be prevented, and medication overuse can be prevented by reducing the use of analgesics. 

In the patient group in which the disease process resulted in consultation, most of the diagnoses were secondary headaches. Although not specified in the table, the consulted departments were cardiology first, then psychiatry, otolaryngology, physical therapy, and rehabilitation. The reasons for consultation were essential hypertension, major depression, generalized anxiety disorder, chronic sinusitis, and musculoskeletal problems. All patients could be easily diagnosed and treated in primary care. The fact that these patients directly seek tertiary care, and even more than one specialty, reduces the accessibility of health services to patients in need. 

The relationship between the family physicians’, neurologists’ and the participants’ own opinions regarding the evaluability of headache in primary care and the diagnostic categories was examined. The family physician’s opinion was that most patients could be evaluated in primary care. Such a high rate of admitting patients with treatable problems to the study’s tertiary care hospital may be due to inadequate family practice. In Yıldız’s dissertation, the opinion of the family physician in primary care was “yes” at a rate of 43.2% [18]. The study by Berberoğlu et al., at different outpatient clinics, showed that the problems of 56.1% of the patients could be solved in primary care [13]. The reason why this rate was higher in our study may be the fact that the study was conducted at a single clinic and on a more specific subject. According to the family physicians’ opinion about treating headaches in primary care, most of those who said “yes” were in the group with a primary headache, which was statistically significant. According to these results, the problems of patients with a primary headache can be resolved in primary care at a higher rate. The “I am not sure” includes patients who are thought to have a primary headache, but who may require additional imaging due to age, other symptoms, or having started medications such as topiramate and carbamazepine, which cannot be prescribed by the family physician. 

In the status of evaluability in primary care, neurologists agree with family physicians. Branch physicians also think that these problems can be resolved in primary care at a high rate. In their study, Yıldız et al. found that the relevant branch physician could evaluate the case in primary care 44.3% of the time [18]. The higher rate in our study may be because the subject is ‘headache’. Most of the patients whose complaints were thought to be resolved were in the group with a primary headache, which was statistically significant. 

The relationship between the patients opinions and those of family physicians and neurologists was found to be statistically significant. A very high proportion of the patients, which the family physician researcher considered to be evaluable in primary care, believed that their problem could not be evaluated in primary care or they were not sure. This is due to the lack of trust in primary care and physicians’ knowledge. For this reason they request examination by a specialist and additional examination requests, since referral chain practice has not been established in Turkey. After patients were informed that their complaints could be solved in primary care, some wanted to change their answer from “no” to “I’m not sure.” From these data, we can conclude that patients do not know how much comprehensive treatment their family physicians can provide. On the other hand, family physicians’ fear of malpractice, patient density, and restrictions on some medications that can be used for headache according to Health Practice Statement rules limit what physicians can do. 

Neurologists reported that all of the patients who stated that their complaints could be evaluated in primary care, almost all of the patients who stated that they were not sure, and almost half of the patients who stated that their problems could not be solved in primary care could in fact be managed in primary care. 

The limitations of our study include the following: the low number of patients, switching to the appointment system due to pandemic measures on the dates when the questionnaire was used and restricting the number of patients, which affected demographic data, the fact that secondary care admittances could not be evaluated because the study was conducted in a tertiary care hospital, the fact that pain intensity and frequency were not included in the evaluation. 

In our study, the opinions of family physicians and neurologists were similar to the patients who applied to tertiary care for the first time with headache complaints about the evaluability in primary care. According to neurologists and the family physicians, most patients who asked for tertiary care could be managed in primary care, and tertiary care was unnecessary for the first-time headache patients.

## Ethical approval

Although our study was not an experimental study on humans, informed consent was obtained from the participants. There were no supporting/funding institutions.
